# Overinterpretation and Overtreatment of Low-Titer Antibodies Against Contactin-Associated Protein-2

**DOI:** 10.3389/fimmu.2018.00703

**Published:** 2018-04-11

**Authors:** Christian G. Bien

**Affiliations:** Epilepsy Center Bethel, Krankenhaus Mara, Bielefeld, Germany

**Keywords:** contactin-associated protein-2 antibodies, depression, cell-based assays, neural antibodies, immunotherapy, diagnostic specificity

## Abstract

Antibodies (abs) against neural or glial antigens have become important diagnostic markers of autoimmune encephalitides. A key requirement for interpretation of any test in clinical medicine is specificity. In this work, a 35-year-old female patient with low-titer contactin-associated protein-2 abs not satisfying clinical criteria of autoimmune encephalitis is reported. The patient had a recurrent depressive disorder and, at the time of the ab study, a moderate depressive episode. Overinterpretation and misinterpretation of patient’s complaints and paraclinical study results fueled the idea of an autoimmune encephalitis. It is suggested to check patients with supposedly positive ab test results critically for clinical criteria, titer cutoffs, and ab-typical epidemiological features like age and sex.

## Background

Immunoglobulin G (IgG) antibodies (abs) against neural or glial antigens have become important diagnostic markers of autoimmune encephalitides or acquired demyelinating central nervous system (CNS) syndromes. A key question in clinical applications is their disease specificity: the clinician needs to be sure that a positive ab result is not an irrelevant finding; e.g., a non-specific product of some other physiological or pathological process, or even a laboratory artifact (that may be unmasked by re-testing the sample). In 2016, two approaches to detect false-positive ab results were described. First, a recent Position Paper authored by international experts delineated a clinical approach for the diagnosis of autoimmune encephalitis that is independent of ab findings and can be used as a plausibility check of a positive ab test result (1). A patient who does not meet the criteria for “possible autoimmune encephalitis,” but is positive for a neural ab, should be carefully studied for alternative explanations for his or her condition. Second, some abs are considered non-specific if they occur below a certain serum titer. This has been suggested or studied for abs against glycine receptors (2), glutamic acid decarboxylase (3) and contactin-associated protein-2 (CASPR2) in the context of the clinical suspicion of autoimmune encephalitis (4). In this work, a patient with low-titer CASPR2 abs not satisfying both specificity criteria described above is reported who repeatedly underwent expensive and potentially harmful treatments.

## Case Presentation

**Figure 1:**
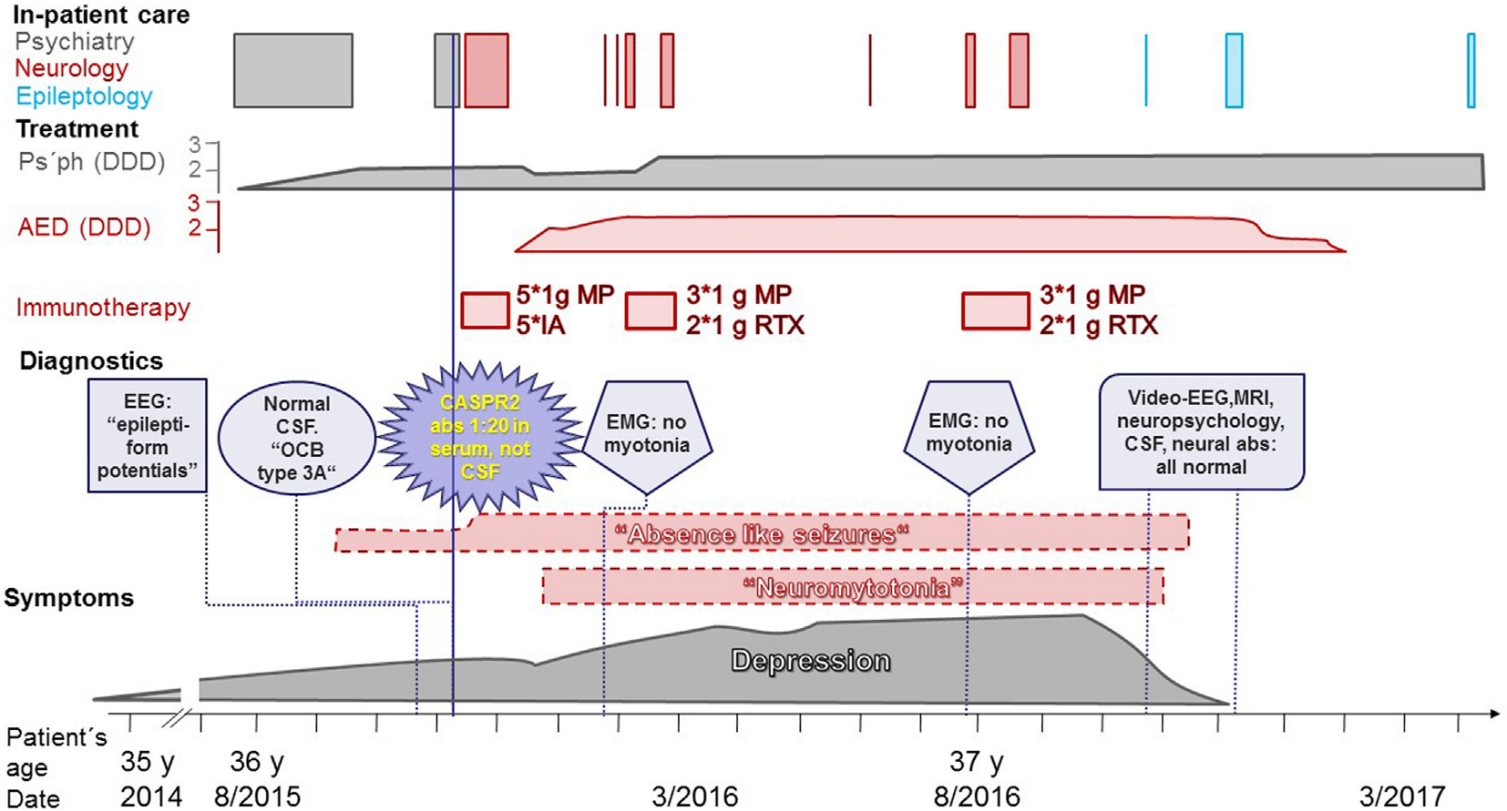
Disease course of the patient. Gray: real symptoms, adequate treatment; red: non-real neurological symptoms (dashed border), treatments in vain; purple: diagnostic findings. Abbreviations: abs, antibodies; AED, antiepileptic drugs; CASPR2, contactin-associated protein-2; CSF, cerebrospinal fluid; DDD, defined daily doses; EEG, electroencephalogram; EMG, electromyogram; IA, immunoadsorptions; MP, intravenous methylprednisolone; MRI, magnetic resonance imaging; Ps’ph, psychopharmacological agents; RTX, rituximab; y, years.

For a graphical presentation of the case, see Figure 1. At the end of 2014, at the age of 35 years, the patient of interest (female of German-Indonesian descent) developed symptoms of depression. No first-degree relatives of the patient suffered from any neuropsychiatric or autoimmune disorders. Some years before, a psychiatrist had diagnosed this patient with a depressive episode and treated her accordingly. She had been trained as a commercial clerk. Due to her high performance and extraordinary commitment, she had been appointed managing director of five companies in Asia with 1,000 employees. After a few years in this position and along with a difficult marriage situation and a toddler, the depressive symptoms evolved. At the age of almost 36 years, self-medication with sedatives and hypnotics was no longer sufficient. Suicidal ideations tormented her. She asked for in-patient admission to a German psychosomatic hospital. The medical report lists the following symptoms: depressive mood, anhedonia, lack of drive, fatigue, concentration and distraction problems, low self-confidence, feelings of guilt, and suicidal thoughts. The diagnosis of recurrent depressive disorder (currently termed moderate depressive episode) was made (ICD-10: F33.1). Despite increasing doses of antidepressant and neuroleptic medication, her mood deteriorated, she complained of memory loss (never formally assessed), and she suffered from a sudden nervous breakdown. She said she would kill herself and her four-year old son. She was transferred to a closed ward of a university psychiatric department.

**Figure 2:**
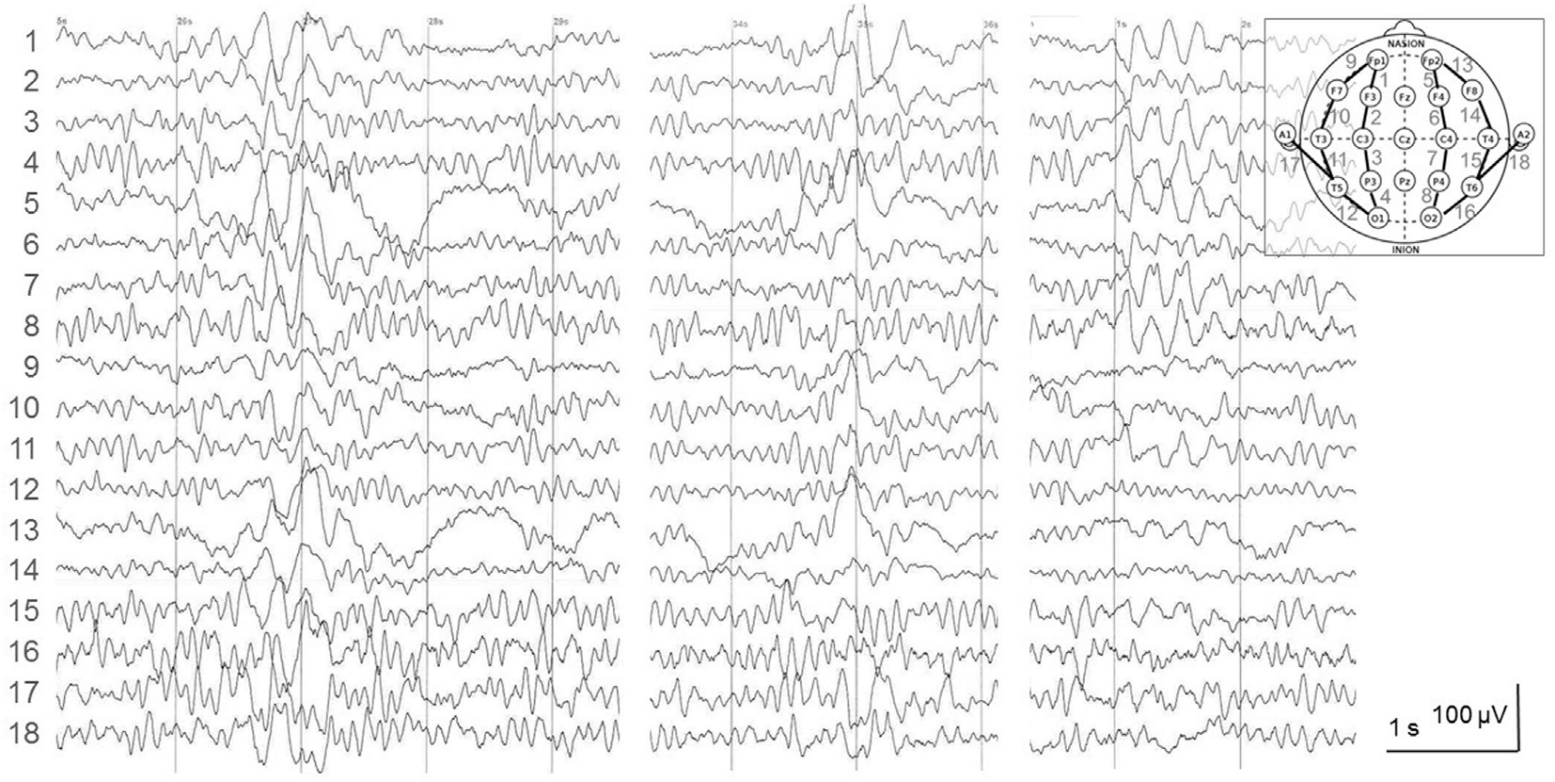
Electroencephalogram (EEG) recorded 12 days prior to the lumbar puncture and the autoantibody diagnostics. The depicted epochs were recorded 30–80 s after onset of hyperventilation (HV), a standard provocation maneuver of epileptiform activity in the EEG laboratory. The EEG was interpreted as follows: “irregular alpha-EEG with right fronto-centro-parieto focus and singular as well as grouped spike-wave complexes with abnormal rhythmizing; strong activation under HV with conduction to the contralateral side and short generalizations.” In fact, this is a normal 9/s-alpha-EEG with physiological high-amplitude slowing under HV. It has been described before that is one typical reason for EEG misinterpretation leading to the erroneous diagnosis of epilepsy (8).

At the psychiatric department, she reported episodes during which she felt detached from the surrounding world. These “bubbles” (as she called them) occurred several times per day. At that time, a doctor from the university’s department of neurology investigated her on the psychiatric ward. The doctor suspected these “bubbles” were epileptic seizures with impaired consciousness, and he ordered a routine electroencephalogram (EEG). The EEG was interpreted as displaying right-hemispheric epileptiform potentials with a tendency to generalize. However, subsequent analysis of the suspicious EEG epochs by the author of this report revealed physiological activity without any potentials suggestive of epileptiform activity (Figure 2). The original EEG reading led to the interpretation of the “bubbles” as focal epileptic seizures. In retrospect, one would probably interpret them as derealization epochs as part of the depressive episode (5). Magnetic resonance imaging (MRI) did not show a potential epileptogenic lesion. Lumbar puncture revealed <1 cell/μl. Neither the IgG index (0.46, normal values <0.7) nor the Reiber diagram suggested an intrathecal IgG synthesis. Isoelectric focusing produced a weak positive result with <4 autochthonous cerebrospinal fluid (CSF) bands, reported by the laboratory as “oligoclonal bands type 3a” (6). Serum and CSF were tested for neural abs using a biochip by Euroimmun (Lübeck, Germany) with human embryonic kidney (HEK) cells expressing the following antigens: *N*-methyl-d-aspartate receptors (NMDAR), α-amino-3-hydroxy-5-methyl-4-isoxazolepropionic acid receptors (AMPAR), leucine-rich glioma inactivated protein 1 (LGI1), CASPR2, and γ-aminobutyric acid-B receptors (GABA_B_R) (7). Serum bound to the CASPR2-expressing cells up to a dilution of 1:20 (endpoint titer). CSF was ab negative. This finding was perceived as a serendipitous turning point in the management of the patient. The previous psychiatric diagnosis was discarded and the diagnosis of anti-CASPR2 autoimmune encephalitis as a causally treatable explanation for the depression and the seizures was made.

The patient was transferred to the university’s neurology department and received five immunoadsorptions, five intravenous (i.v.) boluses of 1 g methylprednisolone (MP) and was started on levetiracetam. She newly reported experiencing pseudohallucinations, but the depression appeared improved; thus, the antidepressant pharmacotherapy was reduced, and the patient was discharged home. Some weeks later, she visited a neurologist in private practice because of recent-onset twitches in her legs. She asked if this may be neuromyotonia. Neither the neurologist’s report nor later medical reports stated that a physician had seen muscle twitches upon physical examination. Myotonic discharges could not be shown by an electromyogram (EMG). Because of ongoing episodes thought to be epileptic seizures, lamotrigine was added. With an increasingly depressed mood, the patient returned to the same neurology department, where neuromyotonia was added to her diagnoses. Three boluses of 1 g i.v. MP plus two infusions of 1 g rituximab (RTX) were administered. After a brief improvement in mood but with ongoing seizures and neuromyotonia, her depression returned. Approximately half a year after the first i.v. MP/RTX course, she received (despite a second negative EMG study) another such treatment.

One year after the diagnosis of anti-CASPR2 encephalitis, with ongoing leg twitching and “bubbles” but improving mood, she visited the author’s clinic (hospital case no. 16810070). During a routine EEG, she had her habitual “bubble” with reduced and slowed responsiveness. An ongoing normal alpha rhythm was recorded (Video S1 in Supplementary Material). A subsequent epileptological in-patient evaluation revealed normal findings in long-term video-EEG, 3Tesla MRI of the brain, and neuropsychological, psychiatric, CSF, and ab investigations. The latter included investigations of CSF and serum by indirect immunofluorescence on mouse brain, on transfected, fixed HEK cells (Euroimmun, Lübeck, Germany), or on immunoblot (Euroimmun, Lübeck, Germany) for abs against the following antigens: NMDAR, LGI1, CASPR2, glycine receptor, AMPAR subunit 2, IgLON family member 5, GABA_B_R, metabotropic glutamate receptor 5, dipeptidyl-peptidase-like protein-6, neuropil abs without further specification, Hu, Ri, Yo, CV2, amphiphysin, Ma2, GAD, recoverin, Sox1, Zic4, Delta/Notch-like EGF-related receptor; for the protocols, see Ref. (4). The depression had remitted. There were no more “bubbles” or leg twitches, and the antiepileptic medication was tapered. An in-patient follow-up with repetition of the aforementioned studies again did not reveal any abnormalities. The patient was discharged with “status post depressive episode” as the only diagnosis. Altogether, the patient spent 48 days in neurological in-patient care in vain, received immunotherapies at a price of approximately 27,000 €, and took antiepileptic drugs for 14 months (2.5 defined daily doses for most of this period). Fortunately, no enduring complications occurred. Written informed consent was obtained from the patient for the publication of this case report.

## Discussion

This patient was erroneously diagnosed in November 2015 with anti-CASPR2 encephalitis, 3 months before the recommendations for a clinical approach to autoimmune encephalitides were published online (1) and 11 months prior to the online publication stating that CASPR2 ab titers (as measured by the Euroimmun assay) in patients diagnosed with autoimmune encephalitis should be much higher than a 1:20 endpoint titer; more specifically, patients with a non-encephalitic MRI (as in this patient) need to have a CASPR2 ab serum titer >1:1,000 to have a >70% likelihood of an autoimmune encephalitis (4).

In retrospect, this patient did not pass the clinical threshold for “possible autoimmune encephalitis” because the psychiatric symptoms did not progress subacutely (1). Also, the previous diagnosis of a recurrent depressive disorder as an alternative diagnosis would have put the diagnosis of autoimmune encephalitis in doubt [see Panel 1 in Ref. (1)]. Additionally, the CASPR2 serum ab titer was too low to permit the diagnosis of an autoimmune encephalitis. Even at the time when the autoimmune encephalitis diagnosis was made, the age and sex of the patient should have raised doubts, since anti-CASPR2 encephalitis is mainly a disorder of men at least 50 years of age (9); this has been confirmed in more recent publications (4, 10, 11).

As an additional (not infrequent) problem, overinterpretation due to overreading of the EEG led to the erroneous diagnosis of epileptiform activity (12), which supported the diagnosis of epilepsy, which was thought to emanate from autoimmune encephalitis. The indeterminate result of “oligoclonal bands type 3a” may have further fueled the idea of a CNS autoimmune process. Meanwhile, it has become clear that less than half of patients with anti-CASPR2 encephalitis who underwent CSF studies studied have intrathecal IgG synthesis (4, 10).

Detection of CASPR2 abs in this patient by a neurologist in the psychiatry department was obviously a striking event. It is tempting to speculate that this contributed to the diagnostic error that this patient had neuromyotonia. Neuromyotonia is a specific feature of Morvan syndrome, which is usually associated with CASPR2 abs (13). The patient reported “leg twitching” only after the detection of the abs. Even though no doctor ever documented the twitches and two EMGs were unable to detect them, the diagnosis of neuromyotonia was noted in the subsequent medical reports (and further corroborated the idea of an anti-CASPR2 encephalitis).

Like a previous report (14), this case study underlines the importance of specificity when making the diagnosis of an autoimmune encephalitis. Clinical criteria (1), titer cutoffs (4), and typical epidemiological features like age and sex may contribute to specific diagnoses of autoimmune encephalitides. Nevertheless, it is possible to detect novel ab-related syndromes. A successful example was the delineation of faciobrachial dystonic seizures in patients with LGI1 abs (15). This example shows that more than one individual is needed to establish such a new association.

## Ethics Statement

This is a retrospective single case study of a patient who was personally treated by the author. Such a publication is covered by the Gesundheitsdatenschutzgesetz (GDSG NRW, German law on healthcare data protection). The patient signed a consent form.

## Author Contributions

There is only one author who did all the work. CGB designed the work; he acquired all data, analyzed, and interpreted them. He wrote the manuscript and provides approval for the publication of the content. He is accountable for all aspects of the work in ensuring that questions related to the accuracy or integrity of any part of the work are appropriately investigated and resolved.

## Conflict of Interest Statement

CGB gave scientific advice to UCB Pharma (Monheim, Germany) and obtained honoraria for speaking engagements from Eisai (Frankfurt, Germany), UCB Pharma (Monheim, Germany), Desitin (Hamburg, Germany), Biogen (Ismaning, Germany), and Euroimmun (Lübeck, Germany). CGB received research support from Deutsche Forschungsgemeinschaft (Bonn, Germany), Gerd-Altenhof-Stiftung (Deutsches Stiftungs-Zentrum, Essen, Germany), Diamed (Köln, Germany), and Fresenius Medical Care (Bad Homburg, Germany). He is a consultant to the Laboratory Krone (Bad Salzuflen, Germany) regarding neural antibodies and therapeutic drug monitoring for antiepileptic drugs.

## Supplementary Material

Video S1 Routine electroencephalogram (EEG) with video recording 1 year after the diagnosis of “anti-CASPR2 encephalitis”. The patient is irregularly blinking with her eyes and produces the typical artifacts. With ongoing blinking, she then has one of her habitual seizures with slowed-down responsiveness. The EEG shows an undisturbed alpha rhythm without signs of an epileptic seizure. These findings speak in favor of a non-epileptic event.
